# Pregnancy Complications and Long-Term Atherosclerotic Cardiovascular Disease Risk

**DOI:** 10.1007/s11883-024-01273-9

**Published:** 2025-01-20

**Authors:** Soniya V. Rabadia, Sarah Heimberger, Natalie A. Cameron, Negeen Shahandeh

**Affiliations:** 1 Department of Medicine, Division of Cardiology, University of California, Los Angeles, Los Angeles, CA USA; 2 Department of Medicine, University of California, Los Angeles, Los Angeles, CA USA; 3 Department of Medicine, Division of General Internal Medicine (N.A.C.), Northwestern University Feinberg School of Medicine, Chicago, IL USA; 4 Department of Medicine, Division of Cardiology, Division of Advanced Heart Failure and Transplant Cardiology, David Geffen School of Medicine at UCLA, Los Angeles, CA USA

**Keywords:** Adverse pregnancy outcomes, Atherosclerotic cardiovascular disease, Hypertensive disorders of pregnancy, Gestational diabetes, Breastfeeding, Sex-specific risk factors

## Abstract

**Purpose of Review:**

Discuss the relationship between pregnancy complications and long-term atherosclerotic cardiovascular disease (ASCVD) risk.

**Recent Findings:**

A large body of research confirms an association between pregnancy complications and increased short and long-term ASCVD risk and seeks to understand mechanisms for these associations. Social determinants of health continue to have a critical impact on the prevalence of adverse pregnancy outcomes (APOs) and long term ASCVD risk. Of the APOs, hypertensive disorders of pregnancy (HDP) are associated with the highest ASCVD risk. Additionally, recent research shows an association between APOs and microvascular coronary heart disease.

**Summary:**

APOs are associated with increased risk of ASCVD, however there is conflicting evidence on whether there is a causal relationship between APOs and ASCVD or if APOs are simply a marker of ASCVD risk. Current ASCVD risk models do not incorporate a history of APOs, therefore it is imperative that healthcare providers take a reproductive health history and account for pregnancy complications when counseling patients on long-term cardiovascular risk. Non-invasive modalities such as coronary artery calcium scoring can be considered as an adjunct, but further research is warranted to determine which patients would benefit most.

## Introduction

Pregnancy is often considered a natural cardiovascular stress test due to the comprehensive physiologic, hemodynamic and metabolic adaptations that must occur to support the developing fetus [1]. Therefore, adverse pregnancy outcomes (APOs) such as hypertensive disorders of pregnancy (HDP), gestational diabetes (GDM), preterm delivery (PTD), and delivering a small-for-gestational-age (SGA) infant, may offer insight into future atherosclerotic cardiovascular disease (ASCVD) risk. In the setting of a growing body of literature describing associations between APOs and long term ASCVD risk, the American Heart Association and American College of Cardiology (AHA/ACC) have recognized APOs as risk enhancing factors for ASCVD [2, 3]. With APOs affecting approximately 1 in 5 births in the United States (US) and rates increasing over recent years, clinicians must have a comprehensive understanding of how to incorporate an obstetric history into ASCVD risk stratification and counseling [4]. In this review, we will describe the current literature in understanding the long term ASCVD risk associated with APOs and provide a summary of recommendations for postpartum management.

## Pathophysiology of APOs and ASCVD

The APOs discussed in this review include HDP, GDM, PTD, SGA and low birth weight (LBW). Their detailed definitions are included in Table 1. Although APOs are phenotypically heterogeneous, they often co-occur, suggesting a shared underlying pathogenesis that may contribute to the development of future ASCVD. The pathogenesis of the majority of APOs is attributed to abnormal placentation, placental ischemia, endothelial dysfunction, and inflammation [5–10]. Acute atherosis in placental spiral arteries has also been shown to occur more frequently in women with PTD, SGA, and preeclampsia, supporting this hypothesis [11]. GDM has a separate pathophysiology and is characterized by beta-cell dysfunction and insulin resistance during pregnancy [12].

**Table 1:** Definition and epidemiology of APOs

APO Type	Definition	Prevalence in the US	Pooled Risk Ratios
Hypertensive Disorders of Pregnancy	HDP: Umbrella term that includes chronic hypertension, gestational hypertension, preeclampsia-eclampsia, and HELLP syndrome. *Chronic hypertension*: Systolic blood pressure (SBP) > 130mmHg or diastolic blood pressure (DBP) ≥ 90mmHg (AHA/ACC) or SBP ≥ 140mmHg or diastolic blood pressure or DBP ≥ 90mmHg (ACOG) before pregnancy or before 20 weeks of gestation *Gestational hypertension (gHTN)*: SBP ≥ 140mmHg or DBP ≥ 90mmHg after 20 weeks of pregnancy when previous blood pressure was normal *Preeclampsia*: multisystem disorder characterized by new onset hypertension and proteinuria or new onset hypertension plus significant end-organ dysfunction with or without proteinuria *HELLP* (H: hemolysis, EL: elevated liver enzymes, LP: low platelets) syndrome: a severe and life-threatening form of preeclampsia that’s characterized by hemolysis, elevated liver enzymes, and low platelet count	HDP: 10.1–15.9%	**HDP** *ASCVD: 1.3–1.6* *CAD: 1.2–2.7* *Stroke: 1.3–1.9* *PAD: 1.6* **gHTN** *ASCVD: 1.4–1.7* *CAD: 1.1–1.7* *Stroke: 1.5–1.6* **Preeclampsia** *ASCVD: 1.5–2.7* *CAD: 1.1–2.2* *Stroke: 1.2-3* *PAD: 1.82*
Gestational Diabetes	Diabetes that develops during pregnancy with no evidence previously and is diagnosed with oral glucose tolerance testing.	7.8%	*ASCVD: 1.3-2* *CAD: 1.1–1.7* *Stroke: 1.5* *PAD: 1.7–2.1*
Preterm Delivery	*Preterm Delivery*: delivery between 20 and 37 weeks of gestation (ACOG) *Subtypes below categorized by World Health Organization (WHO)* *Extremely Preterm*: delivery between < 28 weeks of gestation *Very Preterm*: delivery between 28–32 weeks of gestation *Late Preterm*: delivery between 32–37 weeks of gestation	PTD: 10.38%	*ASCVD: 1.2–2.9* *CAD: 1.5–2.5* *Stroke: 1.3–1.7*
Low Birth Weight/Small for Gestational Age	*Low birth weight: birth weight less than 2.5 kg (kg) (Osterman)* *SGA: birth weight less than 10th percentile*	LBW: 8.60% SGA: 1.5%	**LBW** *ASCVD: 1.3* *CAD: 1.4* **SGA** *Stroke: 1.3–1.5*
Pregnancy Loss	*Miscarriage*: pregnancy loss before 20 weeks gestation. *Stillbirth*: fetal death after 20 weeks gestation. *Medical termination*: medical procedure or medication to terminate a pregnancy.	Stillbirth, Pregnancy Loss, Fetal Death, Ectopic Pregnancy: 2% Medical termination: 1.31%	**Composite of various types of pregnancy loss**: *CAD: 1.1–1.3* *Stroke: 1.1–1.2* **Stillbirth** *CAD: 1.4–1.8* *Stroke: 1.3–1.5*

*ASCVD definition in pooled studies is heterogenous and represents a composite of CAD, stroke, and/or peripheral arterial disease

*Pooled risk ratios are heterogeneous and all studies did not adjust for the same confounding factors

**Table 2:**
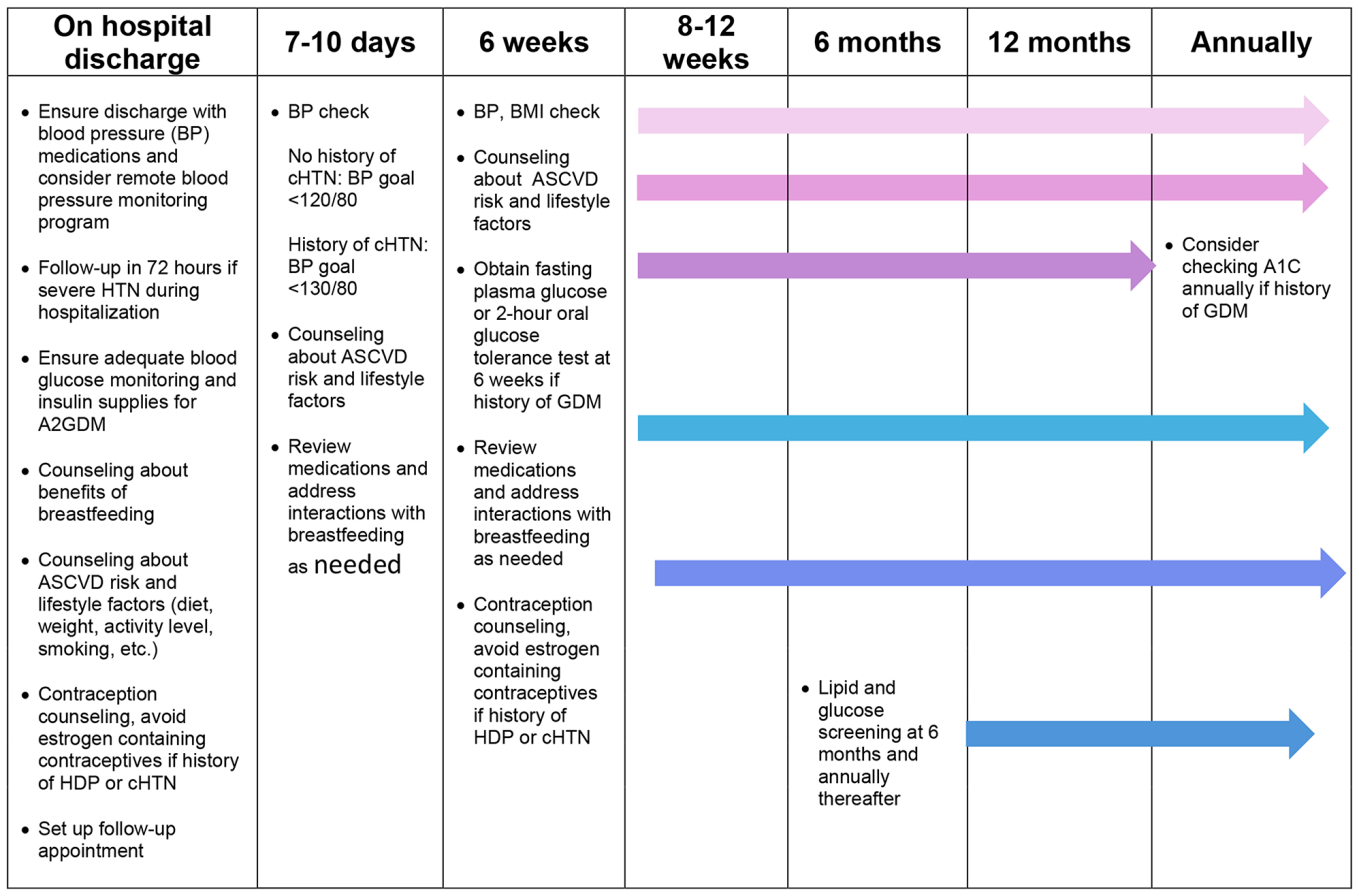
Postpartum management following APOs

**Fig. 1:**
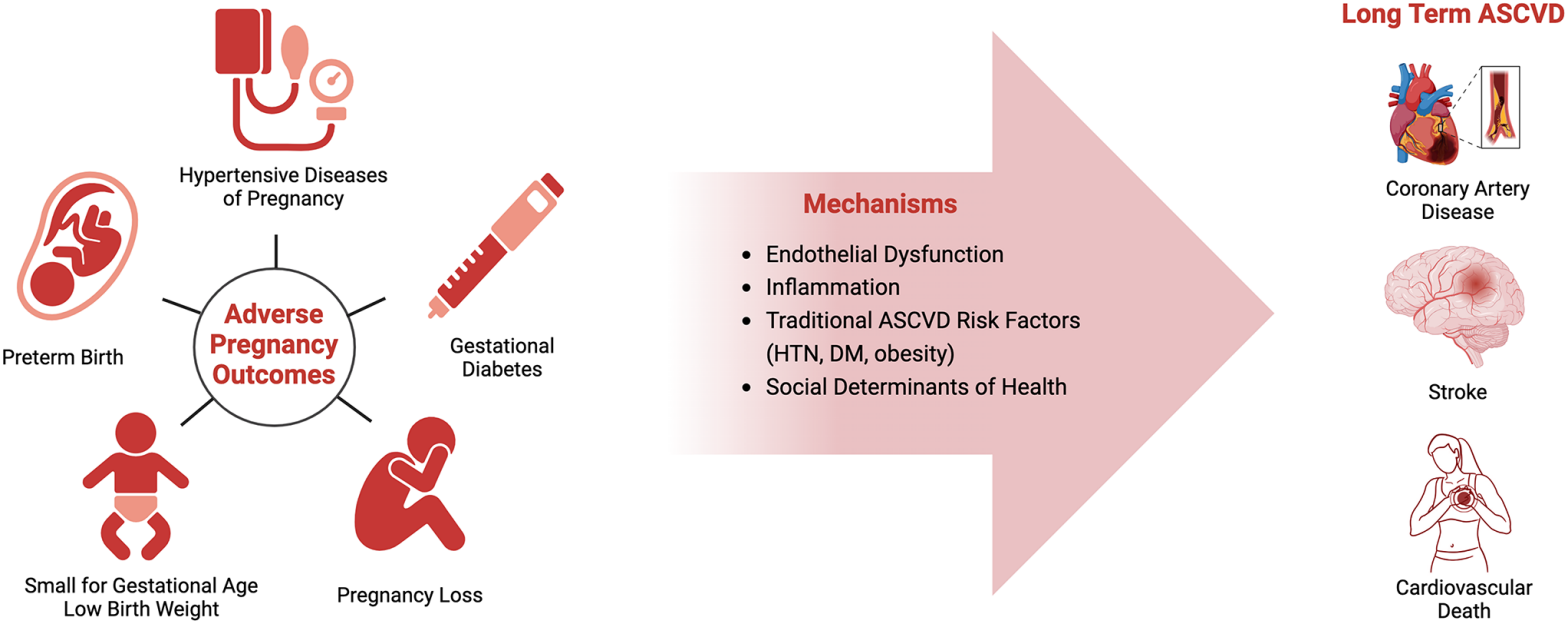
Pregnancy complications and long term ASCVD: proposed mechanisms

Literature has debated the mechanisms by which APOs are associated with long term ASCVD risk (Fig. 1). One leading hypothesis suggests that APOs are mediators (i.e., a causal pathway) of ASCVD [3, 5, 10, 13–15]. APOs are associated with the development of traditional ASCVD risk factors which mediate a portion of the long term ASCVD risk; 49–96% of ASCVD risk mediated by HDP and GDM are attributed to the subsequent development of hypertension (HTN), type 2 diabetes mellitus (DM), and metabolic syndrome [16–19]. Also, APOs contribute to vascular remodeling and inflammation which lead to the development of traditional ASCVD risk factors and ASCVD itself. Preeclampsia has been associated with upregulation of antiangiogenic factors such as soluble fms-like tyrosine kinase-1(sFlt-1), which is also associated with atherosclerosis development [20–22]. Other non-vascular biomarkers have also been implicated with various APOs. Micro-RNA particles identified as miR-222-3p and miR-409-3p have been involved in the development of both GDM and type 2 diabetes mellitus [23]. Additionally, low levels of insulin-like growth factor 1 (IGF-I) have been associated with both decreased fetal growth (which may result in SGA/LBW) and increased ischemic heart disease mortality in the general population [24, 25].

**Fig. 2:**
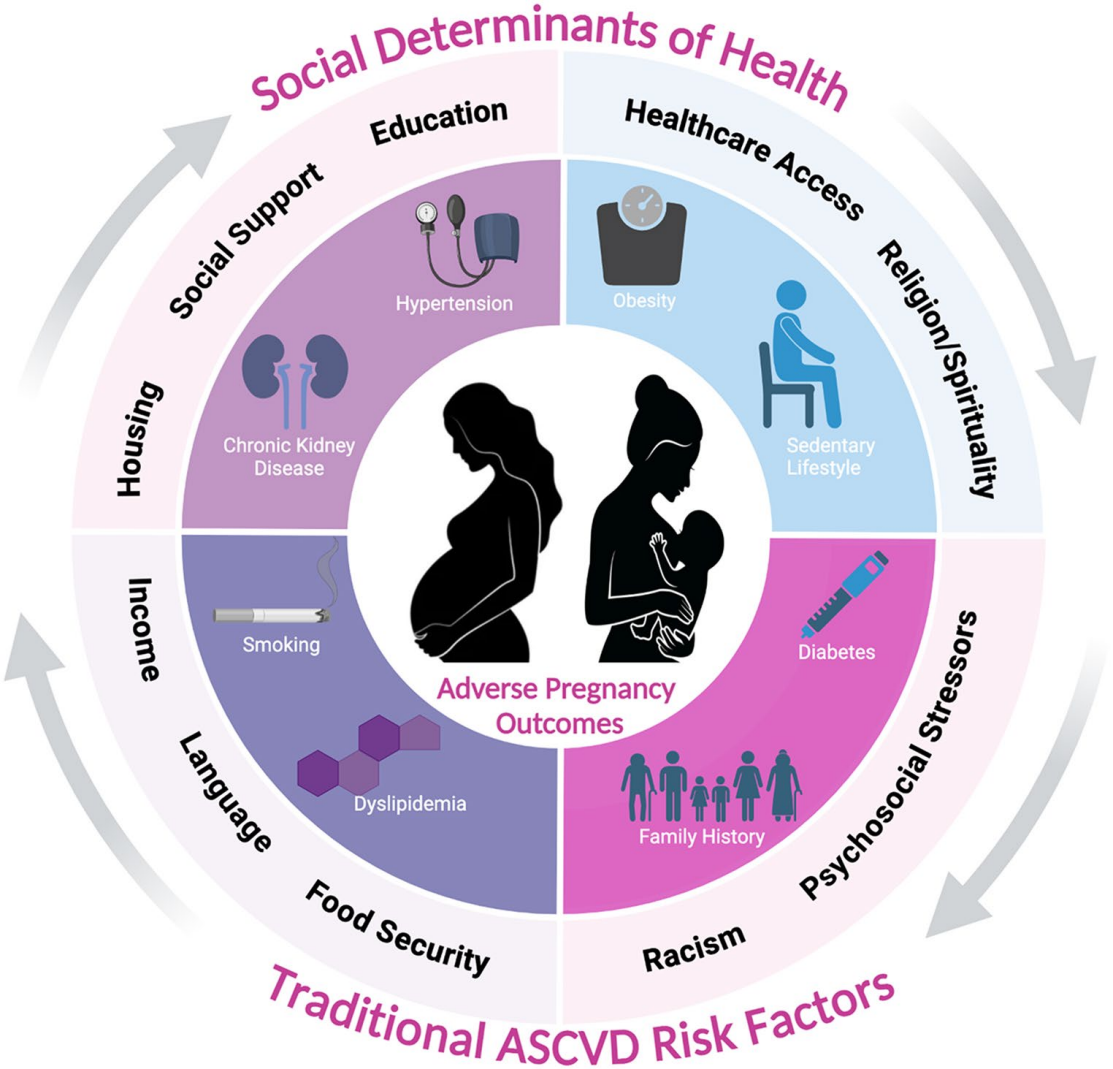
Interplay of social determinants of health and adverse pregnancy outcomes

On the other hand, APOs may represent early markers of ASCVD by unmasking pre-pregnancy risk factors. Shared risk factors for APOs and ASCVD include HTN, DM, dyslipidemia, metabolic syndrome, obesity and smoking [7, 8, 26–28]. Using data from the NuMoM2b Heart Health Study, Khan et al. found that HDP and GDM only mediated 10–13% of the association between pre-pregnancy obesity and ASCVD risk factors up to 7 years postpartum [29]. Further research is needed in this area to understand the complex mechanistic interplay between APOs, biomarkers, genetics, traditional risk factors and future development of ASCVD.

## Social Determinants of Health

Across multiple studies, numerous social determinants of health (SDoH), including but not limited to race/ethnicity, education level, and socioeconomic status, have been associated with increased ASCVD risk (Fig. 2) [30–33]. Similarly, APOs have been shown to disproportionately affect various racial/ethnic groups and individuals from lower socioeconomic status [34–37]. For example, Black women have a 10% higher risk of preeclampsia and 24% higher risk of spontaneous PTD than White women despite adjusting for traditional risk factors and social disadvantage factors [34]. Amongst low income groups, Black and Hispanic women are significantly more likely to develop HDP or GDM compared to other ethnicities [36]. Furthermore, Black, Hispanic, and South Asian women have higher risk of developing type 2 DM after GDM compared to White women [38]. Finally, Black women have a higher rate of mortality within 5 years after developing HDP compared to White women, even after adjusting for socioeconomic and pre-pregnancy metabolic disorders [39]. These findings suggest that the SDoH and structural racism likely contribute significantly to these disparities in maternal health [40, 41]. The AHA has implemented initiatives to address these disparities and provided recommendations to reduce gaps in care and improve maternal outcomes [42]. Further research is needed to elucidate a better understanding of disparities and to create system-wide interventions to lessen these disparities.

## Association of APOs with ASCVD

### Hypertensive Disorders of Pregnancy

HDP, a leading cause of maternal mortality, encompasses a spectrum of disorders including chronic (preexisting) hypertension and a number of pregnancy-associated hypertensive diseases (Table 1) [4, 43–45]. Women with a history of HDP are at increased risk of developing HTN, a known risk factor for ASCVD [16, 46–48]. A 2020 meta-analysis reported that women with HDP have an 18-fold higher risk of developing HTN in the first 6 months postpartum compared to those with normotensive pregnancies. This risk improved but remained 7-fold greater at 1–2 years postpartum [49]. At 15 year follow-up, those with a history of gestational hypertension (gHTN) and severe preeclampsia were approximately 5–6 times more likely to develop HTN than those with normotensive pregnancies, even after adjusting for other APOs [50]. Contemporary studies continue to corroborate these findings and provide evidence that there is additive risk when preeclampsia occurs with other APOs [51, 52].

Although attenuated by the presence of traditional risk factors, studies have also found an independent association between HDP and ASCVD that persists after adjusting for these factors [19, 53]. Among the Women’s Health Initiative (WHI) cohort of postmenopausal women, the risk of ASCVD was 34% greater in those with a history of HDP than those without [53]. Notably, preeclampsia carries greater risk for long-term ASCVD (up to 3-fold) compared to gHTN [19, 54]). Of the types of ASCVD, HDP is most strongly associated with CAD and ischemic stroke [16, 19, 39, 50, 51, 55–59]. Women with a history of HDP have 2–3 times higher risk of developing CAD [39, 51, 55]. Of all the types of HDP, developing recurrent episodes of preeclampsia confers the greatest risk for CAD [50]. With regard to stroke risk, the largest study assessing APOs and ASCVD with greater than 1.3 million participants in the United Kingdom (UK) BioBank reported that HDP was associated with 72% higher risk of ischemic stroke [51]. Moreover, preeclampsia has been associated with up to 3 times higher risk of any type of stroke [54]. Lastly, HDP has been associated with increased risk of PAD [51]. Overall, more severe forms of HDP, HDP superimposed with other APOs, and recurrent HDP are all linked with an increased risk for both ASCVD and traditional risk factor development.

More contemporary studies have also assessed the association between HDP and subclinical atherosclerosis. Preeclampsia is associated with an increased risk of developing coronary artery calcification (CAC) and coronary plaque on cardiac computed tomography (CT) scans [60–62]. Women with a history of preeclampsia are not only more likely to develop CAC than women with normotensive pregnancies, but they do so approximately 5 years earlier [60]. More recently, the CoPenHagen PREeclampsia and CardIOvascUlar diSease (CPH-PRECIOUS) study found that women with early preeclampsia (onset < 34 weeks gestation) had a higher prevalence of coronary atherosclerosis on CT scans 13 years later compared to women with late preeclampsia (onset > 34 weeks gestation). The study adjusted for the presence of concomitant SGA and pre-pregnancy metabolic factors, but not other APOs like PTD [62]. This data may provide an opportunity for early screening for ASCVD in women with history of preeclampsia.

### Gestational Diabetes

GDM refers to the development of diabetes during pregnancy (Table 1) [63–65]. Between 2016 and 2020, the rates of GDM have increased by 30% in the United States [65]. Women with a history of GDM are up to 10 times more likely to develop DM compared to women with normoglycemic pregnancies [66–68]. Moreover, women with A2GDM (medication managed GDM) have up to 4-fold higher risk of subsequent diabetes when compared to women with A1GDM (diet managed GDM). Additional factors that increase risk of future development of DM include higher BMI, family history of DM, multiparity, recurrent episodes of GDM, and advanced maternal age [69, 70].

There is conflicting evidence supporting an independent association between GDM and ASCVD. The Nurses Health II Study found that women with GDM and subsequent DM had a 4-fold higher risk of ASCVD compared to women without GDM, while a history of GDM without subsequent DM was not significantly associated with ASCVD [71]. Other studies report that GDM is associated with 20–60% higher ASCVD risk even when adjusting for future DM [53, 54, 72, 73]. Additionally, the UK Biobank data has shown that at 10 year follow-up, those with history of GDM have a 70% higher risk of having a myocardial infarction, ischemic stroke or PAD individually in an adjusted analysis accounting for traditional risk factors as well as early menopause. There is mixed evidence for an association with stroke risk with some studies demonstrating an association between GDM and stroke, and others concluding that there is not a statistically significant risk [17, 18, 71, 74]. This may be due in part to variations in adjusting for confounding factors and challenges with study designs given heterogenous definitions for stroke. Further studies are needed to clarify whether risk of stroke is attributable to GDM.

Lastly, in regard to subclinical atherosclerosis, GDM has been shown to be associated with the development of CAC and carotid intima-media thickness (CIMT). In the Coronary Artery Risk Development in Young Adults (CARDIA) prospective cohort data, women with GDM had a 2-fold higher risk of developing CAC 15 years after their index pregnancy compared to women without GDM. Even when women did not develop future DM, they still had an independent risk for CAC development. However, the authors reported that when adjusting for developing DM in the future, GDM was not independently associated with CAC development [75]. The CARDIA study also reported that GDM was associated with increased CIMT 20 years after the index pregnancy despite adjusting for numerous confounders including DM [76]. The association of increased CIMT and GDM was also confirmed in a meta-analysis, however, a majority of these studies did not adjust for confounders [77]. With the growing rates of GDM, the early recognition of future DM and ASCVD risk factors is critical for cardiovascular disease prevention.

### Preterm Delivery

PTD is defined as birth between 20 and 37 weeks of gestation (Table 1) [78–80]. It is an important cause of neonatal morbidity and mortality and has a well-described association with long-term maternal ASCVD risk [79, 81, 82]. PTD may be spontaneous or medically indicated, and it often co-occurs with other APOs, including delivering an SGA/LBW infant [83]. Due to this, assessing its independent contribution to ASCVD risk is challenging. Pre-pregnancy risk factors of obesity, HTN, DM, smoking are known to be associated with PTD [7, 8]. Even after adjusting for these pre-pregnancy factors, PTD is associated with a 1.5-fold risk of developing metabolic syndrome up to 25 years after the index pregnancy, with the greatest risk being in the first decade [84]. Studies have reported PTD is associated with up to 3-fold increased risk of ASCVD [53, 54, 85]. When adjusting for LBW, WHI cohort data found that the association between PTD and ASCVD was not statistically significant [53]. However, in a single center study that excluded LBW pregnancies, PTD was associated with 3 times greater risk for ASCVD, and the risk was higher if PTD and LBW occurred concurrently [85].

The risk of CAD and stroke associated with PTD appear to be similar. In a large meta-analysis assessing studies from 2000 to 2017, the overall pooled risk for mortality due to CAD or stroke was 2-fold individually [82]. Medically indicated PTD had greater ischemic heart disease mortality and morbidity compared to spontaneous PTD, likely due to concurrently occurring APOs or other pregnancy complications [86]. Contemporary studies by Crump et al. have expanded upon these findings and found that the risk of ischemic heart disease and stroke associated with PTD persists for 43 years after the index pregnancy with the highest risk present in the immediate decade after pregnancy [87, 88]. They also reported an inverse relationship between gestation length and future ASCVD risk. Extremely preterm deliveries (occurring < 28 weeks gestation) were associated with a 5-fold risk of maternal ischemic heart disease and 5-fold risk for maternal stroke. A large limitation to a majority of studies assessing PTD and ASCVD remains adjusting for confounding APOs like LBW/SGA and distinguishing between the etiology of PTD, whether spontaneous or medically indicated. Therefore, it remains to be determined which factors are driving the increased ASCVD risk associated with PTD. Lastly, given that ASCVD and metabolic syndrome present greater risk within the first 10 years after PTD, clinicians may consider early risk factor screening after PTD.

### Small for Gestational Age/Low Birth Weight

SGA and LBW are separate entities that represent markers of fetal growth and development (Table 1) [80, 89]. Although SGA and LBW are often linked to PTD, LBW has been reported to be independently associated with ASCVD. In the WHI cohort, delivering an infant with LBW was associated with a 12% higher risk for ASCVD even after adjusting for other APOs (including PTD) and traditional risk factors [53]. Delivering an SGA infant is also associated with 30% increased risk of future maternal stroke after adjusting for conventional risk factors [55, 59]. Additionally, offspring birthweight has been inversely associated with long term maternal ASCVD risk [90]. For example, one study that described a 4-fold greater adjusted risk of ischemic heart disease, death or hospitalization associated with delivering infants < 2.5 kg also found that birth weight 2.5–2.99 kg and 3–3.49 kg was associated with 3-fold and 2-fold increased risk, respectively [90]. On the other hand, one study found that women with prior LBW pregnancy and subsequent pregnancies with higher birth weights had some degree of ASCVD risk reduction [91]. It remains uncertain, however, whether the relationship between delivery of LBW or SGA infant and ASCVD is causal, or rather a reflection of preexisting maternal ASCVD risk factors that affect the uteroplacental circulation and result in intrauterine growth restriction.

### Pregnancy Loss

Pregnancy loss is defined as fetal death due to spontaneous miscarriage, stillbirth or termination [92, 93] (Table 1). Although miscarriages are primarily driven by fetal genetic abnormalities, there are shared predisposing factors between miscarriage and ASCVD including obesity and smoking [94, 95]. Miscarriages and stillbirth are also associated with anti-phospholipid syndrome (APLS), which in turn has also been associated with ASCVD [96, 97]. Several studies have assessed the association between pregnancy loss and ASCVD. Although spontaneous miscarriage and stillbirth were not associated with risk of PAD, these pregnancy complications have been associated with CAD and stroke [100]. In a 2022 meta-analysis, authors reported that women with recurrent (2 or more) miscarriages had a 37% increased risk of developing CAD compared to women without a history of pregnancy loss. Moreover, a history of stillbirth portended a 51% increased risk for CAD [98]. Another meta-analysis dedicated to assessing stroke risk reported that miscarriage (hazard ratio [HR] 1.07) and stillbirth (HR 1.38) were associated with increased stroke risk, with greater risk attributed to recurrent events [99].

Contemporary studies support these findings and provide additional information regarding other ASCVD risk and infertility (defined as inability to conceive after 1 year) [95, 100, 101]. Of the types of pregnancy loss, stillbirth continues to be associated with the greatest ASCVD risk with up to 2-fold risk for myocardial infarction and coronary death and up to 2-fold risk for stroke [95, 100]. Furthermore, among nulliparous postmenopausal women with a history of pregnancy loss, history of infertility was associated with 36% higher ASCVD risk compared to women without infertility [102]. An important limitation of these studies, however, is that they do not characterize the variety of physiological and structural causes of infertility. The mechanism behind the greater ASCVD risk associated with stillbirth in comparison to miscarriage is poorly understood. The mechanism may be related to pre-existing maternal factors such as vascular dysfunction and placental factors that contribute more to stillbirth rather than miscarriage. Lastly, a significant limitation of the literature is a lack of standard definitions for fetal loss. More so, the vast majority of studies do not account for pregnancy intention of the women, anomalies, miscarriages, and elective termination.

## Novel Areas of Investigation

### Microvascular Disease

Conventionally, ASCVD is thought to encompass epicardial coronary artery disease and large and medium vessel disease throughout the body. However, as increasing research has expanded our understanding of the coronary microvasculature and its role in cardiovascular health, it is imperative to discuss the potential association of APOs with coronary microvascular disease (CMD). A recent study using myocardial positron emission tomography to assess coronary flow reserve (CFR) found that, compared to non-postpartum controls and patients with normotensive pregnancies, those with preeclampsia with severe features had a lower rest and stress CFR and increased microvascular resistance 4 weeks after delivery, consistent with CMD. This finding supports the hypothesis that preeclampsia is associated with systemic and coronary microvascular dysfunction and provides a potential mechanism to explain the risk of cardiovascular disease associated with preeclampsia in the short-term [103]. Additionally, several studies have found an association between APOs and the long-term risk of CMD. In the WISE-CVD cohort, women with a history of APOs who had ischemic signs and symptoms and non-obstructive coronary arteries had lower CFR by invasive intracoronary testing compared to women without a history of APOs. Limitations of this study include reliance on self-reported histories of APOs and no distinction between which type of APO was experienced [104]. Higher prevalence of CMD via echocardiography has also been demonstrated in individuals with combined histories of preeclampsia and GDM at least 1 year postpartum when compared to individual APO [105]. Countouris et al. expanded upon this and concluded that CMD continued to persist 8–10 years postpartum in individuals with HDP, though these findings were attenuated by the presence of other metabolic factors [106].Therefore, a history of HDP has been associated with both short and long term CMD. Although diagnostic evaluation for CMD remains relatively underutilized and these studies are limited by small sample sizes, these data provide a rationale to consider evaluation for CMD among women with a history of APOs and symptoms of ischemic heart disease.

### Risk Assessment and Postpartum Management

#### Breastfeeding

Breastfeeding is associated with an improved cardiometabolic profile in the short-term as well as decreased long-term ASCVD risk [107, 108]. Women who breastfeed for at least 3 months have lower rates of type 2 DM and HTN later in life, independent of postpartum weight differences [109–111]. Furthermore, longer duration of breastfeeding appears to confer greater benefit [107, 112]. A 2019 meta-analysis found a 30% lower risk of DM and 13% lower risk of HTN among those who breastfeed for at least 12 months [107]. This has been attributed to modulation of cardiometabolic risk factors including decreased visceral fat, waist-hip ratio, fasting serum glucose and triglyceride levels and increased high-density lipoprotein cholesterol levels among those who breastfeed [109–111]. In addition, studies have shown an association between breastfeeding and reduction in both subclinical and clinical ASCVD. In one study, those who never breastfed were more likely to have coronary and aortic calcification and carotid plaque than those who breastfed for 3 or more months [108]. ​​Lastly, breastfeeding is associated with a significantly lower lifetime risk of coronary heart disease, stroke, and CVD mortality, with greater risk reduction seen as the duration of breastfeeding increases within the first 12 months [113, 114].

There is limited data on the benefits of breastfeeding amongst those who experienced APOs. However, breastfeeding has been associated with lower postpartum blood pressures among those who had HDP and lower incidence of metabolic syndrome and type 2 DM amongst those with a history of GDM [29, 106, 112, 115]. Breastfeeding should be seen as an adjunct rather than a replacement for standard cardiovascular risk reduction strategies. For patients that are able to breastfeed, providers should provide resources such as referral to lactation consultants and review medications at each visit to ensure lactation safety. Multiple resources are available for patients and providers to assess medication safety during breastfeeding including LactMed, a National Institute of Health database containing information about drug and chemical safety with regard to breastfeeding [116, 117].

#### Risk Assessment

It is crucial for women who develop pregnancy complications to receive routine follow-up for surveillance and management of ASCVD risk factors. Unfortunately, up to 40% of women do not attend their initial postpartum visit and only 60% of women with a history of APOs or pre-pregnancy ASCVD risk factors receive counseling for cardiovascular risk factor reduction at their postpartum visits [118, 119]. Less than 25% of women with a history of HDP are aware of their 10-year cardiovascular risk [120]. Hence, it is not only imperative to provide comprehensive counseling, but also timely follow-up for women with pregnancy complications. Currently, APOs are recognized as risk enhancers by the AHA/ACC. Although they are not included in the ACC/AHA ASCVD Pooled Cohort equation or the recent Predicting Risk of CVD Events (PREVENT) calculator, several studies have assessed incorporating pregnancy complications such as HDP and PTD in ASCVD risk prediction models. Unfortunately, addition of these factors has not uniformly improved risk classification [84, 121–123].

In Table 2, we provide a schedule for follow-up visits with healthcare providers for risk factor assessment and postpartum management based on AHA/ACC and American College of Obstetrics and Gynecology (ACOG) recommendations [2, 3, 124, 125]. Transition of care between providers during the postpartum period is key to establishing longitudinal care and ensuring comprehensive monitoring of cardiovascular risk factors. Patients who have experienced an APO should have their cardiovascular risk factors and lifestyle factors assessed at each postpartum visit with emphasis on blood pressure checks and BMI assessment. Women with HDP may be considered for remote blood pressure monitoring to enhance postpartum follow-up. Counseling should be provided about long term ASCVD risk. Glucose and lipid assessment should strongly be considered to be completed annually. Lastly, prior studies have reported that subclinical coronary atherosclerosis has been present in women with a history of APOs, especially those deemed low cardiovascular risk by traditional risk factors in the 50–65 years age group [126]. Moreover, women with history preeclampsia have accelerated CAC development between 40 and 50 years of age [60]. Based on these findings, clinicians may consider using CAC scores to assess risk in women who are in the low-intermediate ASCVD risk group with a prior history of APOs. All in all, patients with APOs should undergo consistent follow-up not only in the postpartum period, but also annually to ensure their cardiovascular health is optimized.

## Conclusions

APOs are critical sex-specific factors that should be incorporated into ASCVD risk factor assessment, even though they are not formally a part of standard risk calculators. Gaps in research remain regarding the mechanism behind the association between APOs and ASCVD given the observational nature of the majority of prior research; therefore, more using longitudinal cohorts that span the reproductive life course are needed to understand the interactions between pre-pregnancy cardiovascular health, APOs and ASCVD. Moreover, pregnant intention family planning, and all pregnancy outcomes should be included. Prevention and risk assessment tools are needed to quantify ASCVD risk among people with APOs to guide tailored prevention strategies. Furthermore, studies lack generalizability within the global population and there is space for further assessment of how SDoH contribute to racial disparities in APOs and associated ASCVD risk. Lastly, the pre-pregnancy, postpartum and inter-partum periods are key intervals for counseling, risk factor management, and interventions. Improved clinician and patient education is needed to overall optimize long term ASCVD risk.

## Key References

•• Lewey J, Beckie TM, Brown HL, Brown SD, Garovic VD, Khan SS, et al. Opportunities in the Postpartum Period to Reduce Cardiovascular Disease Risk After Adverse Pregnancy Outcomes: A Scientific Statement From the American Heart Association. Circulation. 2024;149. Latest expert consensus statement on management of cardiovascular risk after adverse pregnancy outcomes.
• Tyrmi JS, Kaartokallio T, Lokki AI, Jääskeläinen T, Kortelainen E, Ruotsalainen S, et al. Genetic Risk Factors Associated With Preeclampsia and Hypertensive Disorders of Pregnancy. JAMA Cardiol. 2023;8:674. Genome wide association study assessing genetic traits of hypertensive disorders of pregnancy and hypertension.
• Lee SM, Shivakumar M, Park JW, Jung YM, Choe EK, Kwak SH, et al. Long-term cardiovascular outcomes of gestational diabetes mellitus: a prospective UK Biobank study. Cardiovasc Diabetol. 2022;21:221. Large, prospective cohort study using UK Biobank data assessing gestational diabetes and multiple ASCVD outcomes with adjusting for traditional risk factors. This study also assessed the degree of ASCVD risk associated with gestational diabetes mediated by other factors.
• Stuart JJ, Tanz LJ, Rimm EB, Spiegelman D, Missmer SA, Mukamal KJ, et al. Cardiovascular Risk Factors Mediate the Long-Term Maternal Risk Associated With Hypertensive Disorders of Pregnancy. J Am Coll Cardiol. 2022;79:1901–13. Nurses Health II longitudinal cohort study assessing the association of hypertensive disorders of pregnancy and cardiovascular disease outcomes. This study also assessed the degree of ASCVD risk associated with hypertensive disorders of pregnancy mediated by other factors.
• Khan SS, Petito LC, Huang X, Harrington K, McNeil RB, Bello NA, et al. Body Mass Index, Adverse Pregnancy Outcomes, and Cardiovascular Disease Risk. Circ Res. 2023;133:725–35. Data from nuMoM2b (Nulliparous Pregnancy Outcomes Study: Monitoring Mothers-To-Be) Heart Health Study showing that adverse pregnancy outcomes only mediate a small proportion of the association between obesity and cardiovascular disease risk factors.
• Miao Q, Guo Y, Erwin E, Sharif F, Berhe M, Wen SW, et al. Racial variations of adverse perinatal outcomes: A population-based retrospective cohort study in Ontario, Canada. Harville EW, editor. PLOS ONE. 2022;17:e0269158. Retrospective cohort study based in Canada that identified greater adverse perinatal outcomes amongst Black women compared to White women.
• Malek AM, Wilson DA, Turan TN, Mateus J, Lackland DT, Hunt KJ. Maternal Coronary Heart Disease, Stroke, and Mortality Within 1, 3, and 5 Years of Delivery Among Women With Hypertensive Disorders of Pregnancy and Pre-Pregnancy Hypertension. J Am Heart Assoc. 2021;10:e018155. Retrospective, racially diverse cohort study of 433, 430 participants from South Carolina. Assessed hypertensive disorders of pregnancy association with short and long term ASCVD outcomes as well as racial/ethnic differences in outcomes.
• Janevic T, McCarthy K, Liu SH, Huyhn M, Kennedy J, Tai Chan H, et al. Racial and Ethnic Inequities in Development of Type 2 Diabetes After Gestational Diabetes Mellitus. Obstet Gynecol. 2023;142:901–10. Retrospective, cohort study in New York City identifying the racial/ethnic differences on risk of type 2 diabetes after history of gestational diabetes.
•• Labgold K, Howards PP, Drews-Botsch C, Dunlop AL, Bryan JM, Ruddock T, et al. Decomposing the Black–White Racial Disparity in Severe Maternal Morbidity Risk: The Role of Hypertensive Disorders of Pregnancy. Epidemiology. 2024;35:94–102. Retrospective analysis of George hospital discharge data to assess the association of hypertensive disorders of pregnancy and severe maternal morbidity risk in non-Hispanic Black and non-Hispanic White patients. If hypertensive disorders of pregnancy was eliminated, only 26% of the Black and White racial maternal morbidity disparity would be decreased.
•• Mehta LS, Sharma G, Creanga AA, Hameed AB, Hollier LM, Johnson JC, Leffert L, McCullough LD, Mujahid MS, Watson K, White CJ. Call to action: maternal health and saving mothers: a policy statement from the American Heart Association. Circulation. 2021 Oct 12;144(15):e251-69. Critical policy statement from the American Heart Association discussing the impact of social determinants of health on maternal cardiovascular outcomes and recommendations on how to reduce disparity gap.
•• Minhas AS, Ogunwole SM, Vaught AJ, Wu P, Mamas MA, Gulati M, et al. Racial Disparities in Cardiovascular Complications With Pregnancy-Induced Hypertension in the United States. Hypertension. 2021;78:480–8. Assessed large dataset from the National Inpatient Sample (NIS) with greater than 10 million Deliveries. Study identified that Black women have the greatest prevalence of preeclampsia. However, although Black women had a higher absolute rate of acute cardiovascular complication after preeclampsia, Asian/Pacific Islander women had the highest relative risk of developing acute cardiovascular complication after preeclampsia after adjusting for confounding factors.
••Rayes B, Ardissino M, Slob EAW, Patel KHK, Girling J, Ng FS. Association of Hypertensive Disorders of Pregnancy With Future Cardiovascular Disease. JAMA Netw Open. 2023;6:e230034. Large genome-wide association study assessing association of hypertensive disorders of pregnancy and coronary artery disease and stroke using FinnGen Study data.
• Xie W, Wang Y, Xiao S, Qiu L, Yu Y, Zhang Z. Association of gestational diabetes mellitus with overall and type specific cardiovascular and cerebrovascular diseases: systematic review and meta-analysis. BMJ. 2022;e070244. Large meta-analysis of 15 studies assessing association between gestational diabetes and cardiovascular disease and adjusted for traditional risk factors.
• Gunderson EP, Sun B, Catov JM, Carnethon M, Lewis CE, Allen NB, et al. Gestational Diabetes History and Glucose Tolerance After Pregnancy Associated With Coronary Artery Calcium in Women During Midlife: The CARDIA Study. Circulation. 2021;143:974–87. Multi-center, prospective cohort study in the United States that reported independent association of coronary calcium development with history of gestational diabetes.
• Crump C, Sundquist J, Sundquist K. Preterm Delivery and Long-Term Risk of Stroke in Women: A National Cohort and Cosibling Study. Circulation. 2021;143:2032–44. Large cohort study in Sweden that identified increased risk of stroke with history of preterm delivery while accounting for traditional risk factors and using cosibling analysis to adjust for genetic and environmental factors.
• Kyriacou H, Al-Mohammad A, Muehlschlegel C, Foster-Davies L, Bruco MEF, Legard C, et al. The risk of cardiovascular diseases after miscarriage, stillbirth, and induced abortion: a systematic review and meta-analysis. Bäck M, editor. Eur Heart J Open. 2022;2:oeac065. Large meta-analysis that assessed the association of various types of pregnancy loss and cardiovascular disease, including induced abortion which had not been included in prior meta-analyses.
• Liang C, Chung H-F, Dobson AJ, Mishra GD. Infertility, Miscarriage, Stillbirth, and the Risk of Stroke Among Women: A Systematic Review and Meta-Analysis. Stroke. 2022;53:328–37. Large meta-analysis assessing association between pregnancy loss and stroke. Miscarriages and stillbirths were associated with stroke, with increased risk for each additional miscarriage and stillbirth.
• Wright CE, Enquobahrie DA, Prager S, Painter I, Kooperberg C, Wild RA, et al. Pregnancy loss and risk of incident CVD within 5 years: Findings from the Women’s Health Initiative. Front Cardiovasc Med. 2023;10:1108286. Women’s Health Initiative cohort study that described cardiovascular risk associated with pregnancy loss in various age groups in the postmenopausal period.
• Murugappan G, Leonard SA, Farland LV, Lau ES, Shadyab AH, Wild RA, et al. Association of infertility with atherosclerotic cardiovascular disease among postmenopausal participants in the Women’s Health Initiative. Fertil Steril. 2022;117:1038–46. Women’s Health Initiative study that described the cardiovascular risk associated with infertility. This reported that infertility with history of no live births was associated with higher cardiovascular risk while adjusting for traditional risk factors.
• Honigberg MC, Economy KE, Pabón MA, Wang X, Castro C, Brown JM, et al. Coronary Microvascular Function Following Severe Preeclampsia. Hypertension. 2024;81:1272–84. Cross-sectional study assessing coronary microvascular function with cardiac positron emission tomography for patients with and without preeclampsia in the postpartum period. Results showed that lower myocardial flow reserve and higher stress coronary vascular resistance in patients with history of preeclampsia.
• Countouris ME, Catov JM, Zhu J, de Jong N, Brands J, Chen X, Parks WT, Berlacher KL, Gandley RE, Straub AC, Villanueva FS. Association of Hypertensive Disorders of Pregnancy With Coronary Microvascular Dysfunction 8 to 10 Years After Delivery. Circulation: Cardiovascular Imaging. 2024;17(5):e016561. Study assessing microvascular dysfunction with echocardiography 8–10 years after delivery. Microvascular dysfunction was associated with history of preeclampsia, however, association strongly driven by metabolic factors.
•• Tschiderer L, Seekircher L, Kunutsor SK, Peters SA, O’Keeffe LM, Willeit P. Breastfeeding is associated with a reduced maternal cardiovascular risk: Systematic review and meta-analysis involving data from 8 studies and 1 192 700 parous women. Journal of the American Heart Association. 2022;11(2):e022746. Large meta-analysis assessing association between breastfeeding and cardiovascular disease. Reported women who breastfed had lower risk of coronary artery disease, stroke, and cardiovascular disease related death.
• Cameron NA, Yee LM, Dolan BM, O’Brien MJ, Greenland P, Khan SS. Trends in Cardiovascular Health Counseling Among Postpartum Individuals. JAMA. 2023;330:359. Cross-sectional analyses describing prevalence cardiovascular health counseling at postpartum visits. Results showed that only 60% of women with cardiovascular risk factors or adverse pregnancy outcomes receiving counseling at postpartum visit.
• Sederholm Lawesson S, Swahn E, Pihlsgård M, Andersson T, Angerås O, Bacsovics Brolin E, et al. Association Between History of Adverse Pregnancy Outcomes and Coronary Artery Disease Assessed by Coronary Computed Tomography Angiography. JAMA. 2023;329:393. Large cross-sectional study in Sweden that assessed association of adverse pregnancy outcomes and coronary atherosclerosis. Women with history of adverse pregnancy outcomes had higher prevalence of coronary atherosclerosis especially in groups deemed to be lower risk.

## Data Availability

No datasets were generated or analysed during the current study.
